# The severity of menopause and associated factors among middle-aged women residing in Arba Minch, DHSS, Ethiopia, 2022

**DOI:** 10.1186/s12905-023-02442-9

**Published:** 2023-05-25

**Authors:** Begosew Misiker, Kefita Kashala, Direslgne Misker

**Affiliations:** 1 Biomedical Science Department, Arba Minch University, Arba Minch, Ethiopia; 2 Biomedical Science Department, Arba Minch University, Arba Minch, Ethiopia; 3 School of Public Health, Arba Minch University, Arba Minch, Ethiopia

**Keywords:** Menopausal symptoms, Arba Minch DHSS, Menopause, The severity of menopause, Middle-aged women, Descriptive crossectional study

## Abstract

**Background:**

Menopause is a common practice in women, and it is recognized as a complete pause of menses for more than twelve months. A decrease in sex hormone levels particularly estrogen in the blood is associated with different types of menopausal symptoms. Those symptoms include different psychological, vasomotor, physical, and sexual symptoms. They are among the major public health problems of middle-aged women. Particularly severe forms of menopausal symptoms are bothersome for middle-aged women. However, little is known about the severity status and associated factors of menopausal symptoms among middle-aged women in the study area.

**Objective:**

The main aim of the present study was to assess the severity of menopausal symptoms and associated factors among middle-aged women residing in Arba Minch DHSS*.*

**Methods and materials:**

Community-based crossectional study was employed. A single population proportion formula was used to determine the sample size. A total of 423 study participants were recruited to conduct the study. A simple random sampling technique was used to employ study participants. Proportional sample size allocation formula was used to allocate study participants in each Kebele of Arba Minch DHSS (demographic and health surveillance site). A menopausal rating scale was used to assess the severity status of Menopausal symptoms. The collected data were analyzed by using SPSS version 20. A descriptive analysis was made to describe the Sociodemographic characteristics of the study participants. Moreover, binary and ordinal logistic regressions were used to identify the factors associated with the severity of menopausal symptoms among middle-aged women. On binary logistic regression variables with *p*-value < 0.25 were eligible for ordinal logistic regression. Variables with a *p*-value < 0.05 were considered statistically significant*.*

**Result:**

The present study revealed that the prevalence of menopausal symptoms was 88.7%. According to the Menopausal rating scale, 91.7% of the study participants were Asymptomatic, 6.6% of them were mild in severity, 1.4% of them were moderate and the remaining 0.23% individuals were severe menopausal symptoms. The most severe menopausal symptom was the sexual problem. The factors that have a significant association with the severity of menopausal symptoms were Age with [AOR = 1.46(95%CI: 1.27–1.64)] and history of chronic disease with [AOR = 2.56(95%CI: 1.78–3.4)] and *p* < 0.001.

**Conclusion and recommendation:**

Generally, menopausal symptoms among middle-aged women were common. Asymptomatic and mild forms are the dominant severity forms of menopausal symptoms. Age and history of chronic diseases have statistically significant associations with the severity of menopausal symptoms. The ministry of health, researchers, and different stakeholders are recommended to be concerned about this neglected issue.

**Supplementary Information:**

The online version contains supplementary material available at 10.1186/s12905-023-02442-9.

## Introduction

Menopause is a normal process in women resulting from the derangement of the estrogen hormone and can be understood as the cessation of menses for more than twelve months [1, 2]. It is considered when the follicular stimulating hormone is greater than 40 UI/l and Estradiol level is less than 20 pg/mL [3]. The depletion of sex hormones particularly estrogen hormone in the blood is associated with different forms of physiological symptoms called Menopausal symptoms [4]. There is a continuous loss of ovarian follicles throughout the reproductive lifespan and a gradual reduction of hormone production by the ovaries, which leads to changes in the length of the menstrual cycle, ultimately to its cessation and the manifestations of different menopausal symptoms [5, 6]. These physiological changes may be associated with different metabolic, cardiovascular, and bone fracture-related complications [7, 8]. Middle-aged women are highly affected by the gradual decrement of sex hormones. Those women are in the period of the menopausal transition and they are vulnerable to different types of menopausal symptoms including hot flush, depression, anxiety and stress, and sexual problems including dyspareunia [9]. The type, severity, and frequency of symptoms vary not only among individuals of different countries but also in the same population with different cultures and ethnicities [10]. In developed countries, vasomotor symptoms like hot flush, vaginal dryness, insomnia, fatigue, and joint pain are more common [11]. A study conducted in Ethiopia reported that a Hot flush was the most common menopausal symptom [12]. Menopausal symptoms may be caused spontaneously due to aging or they can be induced through a medical intervention, that can affect the production of estrogen hormones by the ovary [13].

In some women, menopausal symptoms are so severe that they can affect their social and individual lives [14]. The severity of menopausal symptoms can be assessed by several tools including Menopausal Rating Scale [15]. The lack of information and financial barriers are believed to be the major factor that can affect the use of hormonal replacement therapy(MHT) [9, 10]. It was also revealed that healthcare givers did not prescribe MHT to women who had menopausal symptoms, indicating that they are not concerned about menopausal-related problems and lack of practice on the use of MHT [8, 16–18]. With the prolongation of human life expectancy, females spend more than one‑third of their lifetime in pre-menopause, menopausal transition, and the subsequent post-menopause [19, 20]*.* In Ethiopia, according to the 2007 census more than 9.7 million women were above age 30 [21]*.*

**Fig. 1:**
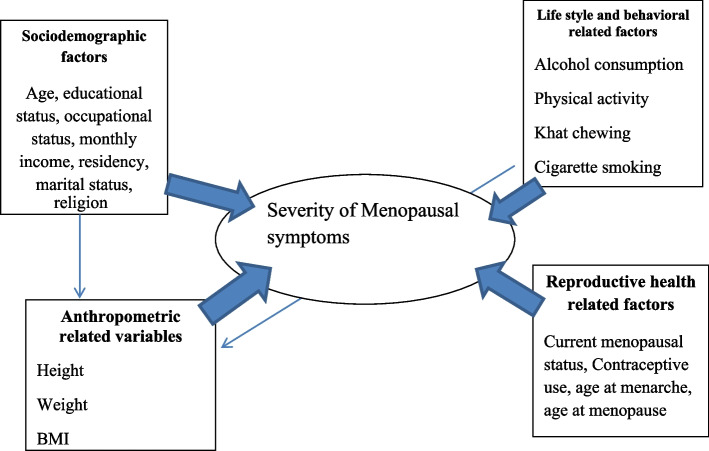
Conceptual framework

Identifying the factors associated with the severity of menopausal symptoms, especially those which are modifiable may be important to reduce the risk of future chronic illness. Different factors are associated with the severity of menopausal symptoms as shown in Fig. 1 [22, 23]. Information about the severity and experience of menopausal symptoms among different racial and ethnic groups is important for healthcare personnel to provide appropriate and specific interventions [15, 22].

## Materials and methods

### Study setting and duration

The study was conducted in Arba Minch Demography and health surveillance site (DHSS). Arba Minch DHSS was established in 2009 GC with 9 Kebele including one town administration and eight rural Kebele. Arba Minch DHSS is found in southern nations, nationalities, and people's regional states. It is located in the Great Rift Valley. The administrative town of this site, Arba Minch, is located about 447 km southwest of Addis Ababa, the capital city of Ethiopia. The site is situated 1285 m above sea level. According to the report from the central statistical agency, 2007, this site has a total population of 164,529, of which about 82,199 were men and 82,330 were women. The study was conducted from June –November 2022.

### Study design

A community-based crossectional study was conducted.

#### Inclusion criteria

All middle-aged women residing in selected villages were included in the study.

#### Exclusion criteria

- ✓ Severely ill individuals
- ✓ Women who have established diagnosis of hyperthyroidism and hypothyroidism
- ✓ Pregnant women

### Sample size determination

- ✓ The sample size was determined by using a single population proportion formula and by considering the prevalence of severe menopausal symptoms among middle-aged women to be 50% because of the absence of a study that was done before in a similar setting on the severity of menopausal symptoms using menopausal rating scale among middle-aged women.
- ✓ *n* = (Zα/2)2pq = (1.96)2 (0.5) (1–0.5) = 384
- ✓ d2 (0.05)2
- ✓ Where, *n* = sample size
- ✓ Zα∕2 = Critical value at 95% confidence interval of certainty (1.96)
- ✓ d = marginal sampling error tolerated
- ✓ *p* = expected prevalence of severe menopausal symptoms among middle-aged women (50%)
- ✓ q = (1-p)
- ✓ By considering a 10%, non-response rate the final sample size was *423.*

### Sampling techniques and procedures

A Simple random sampling technique was employed to select study participants. Households with middle-aged women were coded and a sampling frame was prepared. Then eligible study participants were drawn from the total middle-aged women by using a table of random numbers. Then selected Middle-aged women residing in selected villages were interviewed. Proportional sample size allocation formula was used to allocate study participants in each Kebele of Arba Minch DHSS.

#### Dependent variable

The severity of Menopausal symptoms (coded as asymptomatic, mild, moderate, severe, or very severe).

#### Independent variables

- *Socio-demographic related factors*: Age, education, marital status, occupation, Income, Religion.
- *Lifestyle and behavioral related factors*: cigarette smoking, khat chewing, alcohol intake
- *Anthropometric-related variables*:—BMI, height, weight
- *Reproductive health-related factors*—Contraceptive use, menopausal status, age at menarche, age at menopause.
- *Medically related factors*: History of chronic disease

#### Operational definitions

The Menopause Rating Scale (MRS) comprises 11 items with symptom intensities ranging from 0 = 0 (no symptom/s) to 4.0 (very severe symptoms).

0 = No, the symptom does not occur

1 = Yes, minor or mild symptoms, rarely occurs (monthly):- Duration: last less than 3 min.

2 = Moderate symptom, occurs occasionally (weekly):- Duration: may last up to 5 min.

3 = Severe symptom, occurs frequently (daily):- Duration: last up to 10 min.

4 = Very severe symptom, occurs more frequently: Duration last more than 10 min.

Very severe menopausal symptoms: when they scored > 40 on a menopausal rating scale.

Severe menopausal symptoms: when they scored between35 and 40 on a menopausal rating scale

Moderate menopausal symptoms when they scored between 24–35 on MRS.

Mild menopausal symptoms: when they scored between 12–23 on MRS.

Asymptomatic: when they scored less than or equal to 11 on MRS [15].

*Middle-aged women*: Women who are in the age group of 45–65 years.

*Underweight*: A person having a BMI of < 18.5 kg/m2.

*Overweight*: A person having a BMI of > 24.9 kg/m2.

*Obese*: A person having a BMI of > 30 kg/m2.

*Cigarette-smokers*: Individuals who smoke a cigarette at least once in their lifetime.

*Alcohol-consumer*: Individuals who consume any type of alcohol at least once in their lifetime.

### Data collection tools and procedures

Data were pretested on 22 individuals before the actual data collection and amendments were done on unclear words. Data were collected by using an interviewer-administered structured questionnaire, which is adapted with little modification from WHO. The tool consists of Menopausal Rating Scale, which is an 11-point scale and it is a widely and commonly used tool to assess the prevalence as well as the severity status of menopausal symptoms. It is validated by WHO and it has a minimum score of 0 and a maximum score of 44 [15, 19]. The other part of the tool includes the socio-demographic, economic, medical, reproductive history, and substance-use-related domains of the study subjects. The Data collectors & supervisors were trained and employed by the researcher.

### Anthropometric measurement

The weight and height of the study participants were measured by using a weight-height scale. The participant’s weight was measured without shoes in street clothing and after all heavy materials were removed. Height was measured without shoes. Then BMI was calculated by using SPSS with the formula (weight/height^2^).

### Data management and analysis

The collected data were entered and analyzed by using SPSS version 20. Descriptive analysis was done to describe the Sociodemographic characteristics of the study participant. Frequency distribution tables were used to describe the variables of the study and mean value and standard deviation were used. Binary and ordinal logistic regression analysis was made to identify the factors associated with the severity of menopausal symptoms among middle-aged women. COR and AOR were calculated. The significance level was set at *P* < 0.05 with a confidence interval of 95%. Model fitness and assumption tests were done.

### Ethical consideration

Before the actual data collection, ethical clearance was obtained from the Institutional Review Board of Arba Minch University, College of Medicine and health sciences. Permission was obtained from the Gamo zonal administration office. Oral informed consent was obtained from each study participant. Written consent was also obtained from the legal representatives of illiterate study participants. Participation was voluntary and they were told they can withdraw from the study at any time of data collection. One woman with severe menopausal symptoms was linked to a nearby health institution for further evaluation and management. Possible COVID-19 prevention measures were applied during the interview.

## Result

### Sociodemographic characteristics of study participants

In the present study, 423 study participants participated with a response rate of 100%. The mean age of the study participant was 53.80 with a standard division of 5.786. Among this 285(59.8%) of the study participant were protestant in religion. Of all, 85.3% of the study participants were with a spouse. Of all 315(74.5%) of them were Illiterate in educational status and 340(80.4%) of the study participant were Housewives in occupation. The mean monthly income of the study participant was 1520 with a standard deviation of 1482.05ETB as shown in Table S1.

### Anthropometric and clinical characteristics of the study participant

The mean BMI of the study participant was 27.7 kg/m2 with a standard deviation of 2.9 and 339(80.1%) of the study participant had healthy body mass index. Among all the study participants 83(19.6%) of them have a history of chronic diseases as shown in Table S2.

### Reproductive health status of the study participant

Of 423 study participants 329(77.8%) of them had regular menstrual flow. Of the total study participants, 266(62.9%) of them married at age of 18 and above. Among all 14(3.3%) of them had a history of a hysterectomy and 66(15.6%) of them had a history of contraceptive use as shown in Table S3.

### Substance-use related factors

Among all the study participants 391(92.4%) of them were non-smokers. On the other hand, 112(26.5%) of the study participants consume different types of alcohol as shown in Table S4.

### Severity of menopause

Of the entire study participant, 88.7% of them have at least one type of menopausal symptom. Among the total middle-aged women, 388(91.7%) of them were considered Asymptomatic, 28(6.6%) were mild, 6(1.4%) of the study participants were classified as moderate and only one individual reported severe menopausal symptoms as determined by the menopausal rating scale. In the present study, there are no very severe menopausal symptoms as shown in Table S5.

### The Severity of individual menopausal symptoms

Among the common individual menopausal symptoms that were reported by study participants, the most severe menopausal symptom was sexual problems with 19(4.5%) very severe symptoms and 20(4.7%) severe symptoms as shown in Table S6.

### Factors affecting the severity of menopausal symptoms of middle-aged women

In the present study among the Sociodemographic variables that were analyzed by binary logistic regression; occupational status, age, marital status, and educational level were eligible for ordinary logistic regression. However, on Ordinary logistic regression the Sociodemographic variables that have a significant association with the severity of menopausal symptoms were only age with [AOR = 1.46(95% CI: 1.27–1.64)] and *P* < 0.001). Being older age is more prone than the lower age groups to be affected by severe menopausal symptoms as shown in Table S7.

### Reproductive health-related factors

Regarding the reproductive health-related factors, menstrual status and contraceptive history were associated with the severity of menopausal symptoms on binary logistic regression. However, no variable has a statistically significant association with the severity of menopause on Ordinal logistic regression as shown in Table S8.

### Substance use, anthropometric, and medical-related factors

On Binary logistic regression of the substance use, anthropometric, and medical-related factors; history of cigarette smoking, BMI, and presence of a history of chronic diseases were eligible for ordinal logistic regression. On the other hand, there are no substance use and anthropometric-related variables that were significantly associated with the severity of menopausal symptoms on ordinal logistic regression. Regarding medical-related factors perceived history of chronic disease was significantly associated with the severity of menopause on ordinal logistic regression with [AOR = 2.56(95% CI: 1.78–3.4) and *p* < 0.001]. Middle-aged women with a perceived history of chronic diseases are prone to severe forms of menopausal symptoms as shown in Table S9.

## Discussion

This is a community-based study, aimed to determine the severity and associated factors of menopausal symptoms among middle-aged women residing in Arba Minch, DHSS, Ethiopia. In the present study, the prevalence of menopausal symptoms is 375(88.7%). This study is in line with a study done among Saudi women by Alaa Yasen Al-Olayet et.al who reported that the prevalence of menopausal symptoms was ranging from 80.7–82.8% based on their menopausal status [13]. The higher prevalence of menopausal symptoms among study participants was due to the depletion of the ovarian hormone estrogen and progesterone as a result of the age-associated failure of ovarian follicles [24]. The finding of the present study was higher than a study done in Baltimore, USA by Rima J. et al. [25]. The difference may be due to differences in the study population, socioeconomic status, and even menopausal status of the study participant. The finding of the present study regarding the prevalence of menopausal symptoms was also higher than a study done in Taiyuan by Jian-Ping Zhang et al. who reported that the prevalence of menopausal symptoms was 49.8% [26]. The difference may be due to differences in geographic location, Sociodemographic characteristics, and socioeconomic status of the study participants.

### Severity status of menopausal symptoms

The most severe menopausal symptom that is reported by middle-aged women residing in Arba Minch DHSS was sexual problems with a frequency of 20(4.7%) and 19(4.5%) severe and very severe menopausal symptoms respectively. The finding of the present study was in line with a study done in Korea by Yim et.al who showed that severe to very severe sexual problem was reported in 27.1% of study participants [23]. The reason behind the presence of a severe sexual problem in middle-aged women is the gradual depletion of the hormone estrogen. This is a very important hormone that is crucial for vaginal lubrication and prevents pain during sexual intercourse [19]. This hormone is also very important to prevent vaginal atrophy and dryness. The finding of the present study regarding the severity of individual symptoms is inconsistent with a study done in Pakistan, which reported that psychological symptoms were the most common and severe forms of menopausal symptoms [27]. A study done in Asia also documented that vasomotor symptoms were more common and severe among middle-life women [18]. The difference may be due to differences in the Sociodemographic and socioeconomic status of the study participant.

The present study also revealed the severity status of menopausal symptoms among middle-aged women residing in Arba Minch DHSS by using WHO validated menopausal rating scale. Among the total study participants, 388(91.7%) of cases were asymptomatic The finding of the present study is inconsistent with a study done in Italy by Concetta Maria Vaccaro et al. who reported that only 12.9% of women report asymptomatic severity from menopause [17]. Of all, 28(6.6%) cases were mild.

The frequency of moderate severity status of menopausal symptoms was reported to be six (1.4%). The prevalence of severe menopausal symptoms among middle-aged women was one (0.23%). The finding of the present study is supported by a study done in Iran by Marzieh et.al who reported that severe menopausal symptoms are only reported in three individuals [10]. The lower prevalence of severe menopausal symptoms in the present study may be due to the un-ability to recognize and remember severe menopausal symptoms. The finding of the present study regarding the severity of menopausal symptoms was lower than a study done by Rasha in Egypt, which revealed that the prevalence of severe menopausal symptoms was 17.1% [28]. This finding is also inconsistent with a study done among East Asian women by Qi Yu et al. Reported that the prevalence of moderate to severe vasomotor menopausal symptoms was 55% [18]. This difference in the severity of menopausal symptoms may be due to the difference in the Sociodemographic and socioeconomic characteristics of the study participant; the difference in the tool used to evaluate the severity status of menopausal symptoms of the study participants, and it could be due to the difference in the sample size recruited in the study.

### Factors affecting the severity of menopause

In the present study, it was shown that age has a statistically significant association with the severity of menopausal symptoms. Higher-age individuals are more prone to develop severe forms of menopausal symptoms with [AOR = 1.46(95% CI: 1.27–1.64)] and *P* < 0.001). The finding of the present study was supported by a study done in Iran [22]. This also goes in line with a study done in china by Wenhua Liu et al. reported that age is associated with the frequency and severity of menopause [29]. The main reason for this is that as age increases the estrogen hormone depletes in a significant amount [14, 30]. Due to this severe forms of menopausal symptoms may occur. The finding of the present study is also supported by a study done in Korea by Gyeyoon Y et al. [23].

The finding of the present study also revealed that the presence of a history of chronic disease was significantly associated with the severity of menopausal symptoms with [AOR = 2.56(95% CI: 1.78–3.4)] and *p* < 0.001. Those middle-aged women who have a history of chronic disease were 2.56 times more prone to develop severe forms of menopausal symptoms than individuals with no history of chronic disease. The finding of the present study was in line with a study done in Taiwan [31]. The reason is that middle-aged women with menopausal symptoms may develop chronic diseases and menopausal women with chronic diseases may result in severe symptoms. Low levels of estrogen can raise the risk of heart disease and cardiovascular disease risk factors. Some diseases like autoimmune disorders can affect the ovaries and can exacerbate the low levels of estrogen in menopausal women. A study done by Genazzani AR et al. among postmenopausal women reported that hormonal therapy would be able to improve severe and bothersome symptoms, it is very important in cardiovascular risk reduction, an increase in bone mineral density, and a reduction in bone fracture risk [16].

In the present study menstrual history, marital status, monthly income, Body mass index, and religion have no significant association with the severity of menopause. The finding of the present study was against research done in Egypt [28]. The finding of the present study is also inconsistent with a study done among Chinese women by Zheren Huang et al. reported that educational level is significantly associated with the severity of menopausal symptoms [29]. The reason may be due to similarities in lifestyles of the study participant and also the small sample size recruited in the study.

### Conclusion and recommendation

The present study revealed that menopausal symptoms are common among middle-aged women. Regarding the severity of menopause, asymptomatic and mild forms of menopausal symptoms are common among study participants. Age and history of chronic diseases were significantly associated with the severity of menopausal symptoms. Future researchers are recommended to do large-scale and longitudinal studies on the severity of menopausal symptoms.

### Limitations of the study

Failure to do biochemical tests like the level of estrogen hormone and bone mineral density, which are very important tests that are crucial to determining the cause and severity of menopausal symptoms. Being a crossectional study design may not show the typical cause-and-effect relationship between the factors and the outcome variable.

## Supplementary Information

**Additional file 1:** **Table 1.** Sociodemographic characteristics of middle aged women residing in Arba Minch, DHSS, Ethiopia 2022. **Table 2.** Anthropometric and clinical characteristics of middle aged women residing in Arba Minch DHSS, Ethiopia, 2022. **Table 3.** Reproductive health status of middle aged women residing in Arba Minch DHSS, Ethiopia, 2022. **Table 4.** Substance use related characteristics of study participants residing in Arba Minch DHSS, Ethiopia, 2022. **Table 5.** Prevalence and severity of menopausal symptoms among middle-aged women residing in Arba Minch DHSS, 2022. **Table 6.** Prevalence and severity of individual menopausal symptoms among middle aged women residing in Arba Minch DHSS, Ethiopia, 2022. **Table 7.** Binary and ordinal logistic regression of Sociodemographic factors with the severity of menopausal symptoms among middle-aged women, Arba Minch DHSS, Ethiopia, 2022. **Table 8.** Binary and ordinal logistic regression of reproductive health related factors among middle aged women, Arba Minch, Ethiopia, 2022. **Table 9.** Binary and ordinal logistic regression of substance use, anthropometric and medical related factors of middle-aged women residing in Arba Minch DHSS, Ethiopia, 2022. **Table 10.** frequency of individual menopausal symptoms among middle-aged women residing in Arba Minch DHSS, Ethiopia. 2022.

## Data Availability

The dataset used during the present study is available from the corresponding author upon reasonable request.

## Abbreviations

DHSS: Demographic and health surveillance site
BMI: Body mass index
MRS: Menopausal rating scale
