# Supplementary material for: The severity of menopause and associated factors among middle-aged women residing in Arba Minch, DHSS, Ethiopia, 2022
Source: BMC Womens Health. 2023 May 25;23:287. doi: 10.1186/s12905-023-02442-9 (PMC10211301; doi:10.1186/s12905-023-02442-9)
Supplement: Supplementary file 1 — Additional file 1: Table 1. Sociodemographic characteristics of middle aged women residing in Arba Minch, DHSS, Ethiopia 2022. Table 2. Anthropometric and clinical characteristics of middle aged women residing in Arba Minch DHSS, Ethiopia, 2022. Table 3. Reproductive health status of middle aged women residing in Arba Minch DHSS, Ethiopia, 2022. Table 4. Substance use related characteristics of study participants residing in Arba Minch DHSS, Ethiopia, 2022. Table 5. Prevalence and severity of menopausal symptoms among middle-aged women residing in Arba Minch DHSS, 2022. Table 6. Prevalence and severity of individual menopausal symptoms among middle aged women residing in Arba Minch DHSS, Ethiopia, 2022. Table 7. Binary and ordinal logistic regression of Sociodemographic factors with the severity of menopausal symptoms among middle-aged women, Arba Minch DHSS, Ethiopia, 2022. Table 8. Binary and ordinal logistic regression of reproductive health related factors among middle aged women, Arba Minch, Ethiopia, 2022. Table 9. Binary and ordinal logistic regression of substance use, anthropometric and medical related factors of middle-aged women residing in Arba Minch DHSS, Ethiopia, 2022. Table 10. frequency of individual menopausal symptoms among middle-aged women residing in Arba Minch DHSS, Ethiopia. 2022. [file 12905_2023_2442_MOESM1_ESM.doc]

Table 1. Sociodemographic characteristics of middle aged women residing in Arba Minch, DHSS, Ethiopia 2022(n=423).

| Variable | Category | Frequency | Percentage (%) |
| --- | --- | --- | --- |
| Age | Mean | 53.80±5.786 | |
|  | <55 | 270 | 63.8 |
|  | ≥55 | 153 | 36.2 |
| Marital status | With Spouse | 361 | 85.3 |
|  | Without spouse | 62 | 14.7 |
| Religion | Orthodox | 156 | 36.9 |
|  | Protestant | 253 | 59.8 |
|  | Other | 14 | 3.3 |
| Educational status | Illiterate | 315 | 74.5 |
|  | Primary and above | 108 | 25.5 |
| Occupational status | Employment | 64 | 15.1 |
|  | Housewife | 340 | 80.4 |
|  | Un-employed | 19 | 4.5 |
| Income | Mean | 1520±1482.5 |  |

*other; Muslim=7 and catholic

##### Table 2. Anthropometric and clinical characteristics of middle aged women residing in Arba Minch DHSS, Ethiopia, 2022(n=423).

| Variables | Category | Frequency | Percent |
| --- | --- | --- | --- |
| BMI | Mean | 22.709±2.908 | |
|  | Underweight | 13 | 3.1 |
|  | Normal | 339 | 80.1 |
|  | Above normal | 71 | 16.8 |
|  | Total | 423 | 100 |
| History of chronic disease | Yes | 83 | 19.6 |
|  | NO | 340 | 80.4 |
|  | Total | 423 | 100 |

##### Table 3. Reproductive health status of middle aged women residing in Arba Minch DHSS, Ethiopia, 2022.

| Variables | Category | Frequency | Percent |
| --- | --- | --- | --- |
| Age at marriage | <18 | 157 | 37.1 |
|  | ≥18 | 266 | 62.9 |
|  | Total | 423 | 100 |
| Menstrual history | Regular | 329 | 77.8 |
|  | Irregular | 94 | 22.2 |
|  | Total | 423 | 100 |
| History of hysterectomy | Yes | 14 | 3.3 |
|  | No | 409 | 97.7 |
| Contraceptive history | Yes | 66 | 15.6 |
|  | No | 357 | 84.4 |

##### Table 4. Substance use related characteristics of study participants residing in Arba Minch DHSS, Ethiopia, 2022(n=423).

| Variable | Category | Frequency | Percent |
| --- | --- | --- | --- |
| Cigarette smoking | Yes | 32 | 7.6 |
|  | No | 391 | 92.4 |
| Khat chewing | Yes | 0 | 0 |
|  | No | 423 | 100 |
| alcohol drinking | Yes | 112 | 26.5 |
|  | No | 311 | 73.5 |

##### Table 5. Prevalence and severity of menopausal symptoms among middle-aged women residing in Arba Minch DHSS, 2022 (n=423).

| Variable | Category | Frequency | Percent |
| --- | --- | --- | --- |
| Presence of menopausal symptoms | Yes | 375 | 88.7 |
|  | No | 48 | 11.3 |
| Severity of menopause | Asymptomatic | 388 | 91.7 |
|  | Mild | 28 | 6.6 |
|  | Moderate | 6 | 1.4 |
|  | Severe | 1 | .23 |
|  | Very severe | 0 | 0 |

##### Table 6. Prevalence and severity of individual menopausal symptoms among middle aged women residing in Arba Minch DHSS, Ethiopia, 2022(n=423).

| Symptoms | Hot flush  N (%) | Heart problem n(% | Bladder problem n (%) | Vaginal  Dryness | Anxiety | Depression | Irritability | Joint pain | Sexual pro | Sleep pro | Exhaustion |
| --- | --- | --- | --- | --- | --- | --- | --- | --- | --- | --- | --- |
| Severity |
| None | 270(63.8) | 268(63.3 | 341(80.6 | 306(72.3 | 394(93.1 | 262(61.9) | 257(60.7) | 263(62.2 | 267(63.1 | 248(58.6) | 390(92.2 |
| Mild | 100(23.6 | 93(21.9) | 44(10.4) | 45(10.6 | 11(2.6) | 98(23.2) | 96(22.7) | 97(22.9 | 60(14.2 | 93(21.9) | 5(1.2) |
| Moderate | 39(9.2 | 43(10.2) | 23(5.4) | 45(10.6 | 11(2.6 | 40(9.4) | 47(11.1 | 47(11.1 | 57(13.5 | 64(15.1) | 13(3.07) |
| Severe | 9(2.1 | 12(2.8) | 10(2.4) | 12(2.8 | 7(1.6) | 14(3.3) | 14(3.3 | 10(2.4) | 20(4.7) | 17(4.0) | 11(2.6) |
| Very severe | 5(1.2) | 7(1.6) | 5(1.2) | 15(3.5 | 0 | 9(2.1) | 9(2.1) | 6(1.4) | 19(4.5) | 1(0.2) | 4(0.94) |

##### Table 7. Binary and ordinal logistic regression of Sociodemographic factors with the severity of menopausal symptoms among middle-aged women, Arba Minch DHSS, Ethiopia, 2022 (n=423).

| Variable | Category | Asymptomatic  n(%) | Mild  n (%) | Moderate  n(%) | Severe  n(%) | COR  95%-CI | AOR  95%CI |
| --- | --- | --- | --- | --- | --- | --- | --- |
| Age | <55 | 260(67.01) | 7(25) | 39(50) | 0 | 1 | 1 |
|  | ≥55 | 128(32.99) | 21(75) | 3(50) | 1(100) | **1.287(1.54-8.4)** | **1.46(1.27-1.64)** |
| Religion | Orthodox | 152(39.2) | 4(14.3) | 0 | 0 | 0.5(0.53-1.7) | 1.36(-3.2-0.55) |
|  | Protestant | 224(57.7) | 22(78.6) | 6(100) | 1(100) | 1.2(1.05-1.86) | 0.26(-1.4-1.6) |
|  | Other | 12(3.09) | 2(7.1) | 0 | 0 | 1 | 1 |
| Educational status | Illiterate | 286(73.7) | 25(89.3) | 4(66.7) | 0 | 1.021(0.6-1.7) | 0.37(0.56-1.3) |
|  | Primary and above | 102(26.3) | 3(10.7) | 2(33.3) | 1(100) | 1 | 1 |
| Occupational status | Employment | 51(13.14) | 11(39.3) | 1(16.7) | 1(100) | 0.32(-1.2-2.43) | 1.2(-1.03-3.3) |
|  | Housewife | 319(82.2) | 16(57.1) | 5(83.3) | 0 | 0.031(0.74-1.4) | 0.2(-2.3-1.9) |
|  | Un-employed | 18(4.6) | 1(3.6) | 0 | 0 | 1 | 1 |
| Marital status | With spouse | 335(86.3) | 20(71.4) | 5(83.3) | 0 | 1 | 1 |
|  | Without spouse | 53(13.66) | 8(28.6) | 1(16.7) | 1(100) | 0.04(0.01-0.13) | 0.064(-0.86-0.99) |
| Family monthly income | <500 | 168(94.9) | 8(4.5) | 1(0.6) | 0 | 0.32(1.9-2.4) | -.430(-1.4-0.5) |
|  | 500-1000 | 28(80) | 4(11.4) | 3(8.6) | 0 | 1.54(1.76.2-2.4) | 1.075(0.7-2.1) |
|  | 1000-1500 | 43(93.5) | 2(4.3) | 1(2.1) | 0 | 0.32(-0.2-1.46) | -.136(-1.4-1.2) |
|  | 1500-2000 | 31(86.1) | 4(11.1) | 1(0.3) | 0 | 0.69(2.2-2.93) | .700(0.4-1.8) |
|  | >2000 | 118(92.2) | 10(7.8) | 0 | 1 | 1 | 1 |

*Bold indicates statistically significant variable

##### Table 8. Binary and ordinal logistic regression of reproductive health related factors among middle aged women, Arba Minch, Ethiopia, 2022(n=423).

| Variable | Category | Asymptomatic  n(%) | Mild  n (%) | Moderate  n(%) | Severe  n(%) | COR  95%-CI | AOR  95%CI |
| --- | --- | --- | --- | --- | --- | --- | --- |
| Menstrual history | Regular | 307(79.1) | 17(60.7 | 5(83.3) | 0 | 1 | 1 |
|  | Irregular | 81(20.9) | 11(39.3) | 1(16.7) | 1(100) | **2.056(1.23-3.54)** | 0.8(-1.6-0.11) |
| Age at marriage | <18 | 150(38.6) | 4(14.3) | 3(50) | 0 | 1.06(0.99-1.1) | -1.15(-2-0.26) |
|  | ≥18 | 238(61.3) | 24(85.7) | 3(50) | 1(100) | 1 | 1 |
| Contraceptive history | Yes | 61(15.7) | 2(7.1) | 2(33.3) | 1(100) | 0.19(0.04-0.8) | .12(-1.1-0.88) |
|  | No | 327(84.3) | 26(92.9) | 4(66.6) | 0 | 1 | 1 |
| History of hysterectomy | Yes | 10(2.6) | 2(7.1) | 2(33.3) | 0 | 0.603(0.39-5.1) | 1.153(0.08-2.3) |
|  | No | 378(97.4) | 26(92.9) | 4(66.6) | 1(100 | 1 | 1 |

##### Table 9. Binary and ordinal logistic regression of substance use, anthropometric and medical related factors of middle-aged women residing in Arba Minch DHSS, Ethiopia, 2022(n=423).

| Variable | Category | Asymptomatic  n (%) | Mild  n(%) | Moderate  n(%) | Sever  n(%) | COR  95%-CI | AOR  95%CI |  |
| --- | --- | --- | --- | --- | --- | --- | --- | --- |
| Cigarette smoking | Yes | 32(8.2) | 0 | 0 | 0 | 2.056(0.031-1.77) | 13.5(0.01-13.6) |  |
|  | NO | 356(91.8) | 28(100 | 6(100) | 1(100 | 1 | 1 |  |
| Alcohol consumption | Yes | 104(26.8) | 6(21.4) | 2(33.3) | 0 | 0.495(0.285-1.3 | -0.49(-1.4-0.46) |  |
|  | No | 284(73.2) | 22(78.6) | 4(66.6) | 1(100 | 1 | 1 |  |
| BMI | Underweight | 9(2.3) | 2(7.1) | 2(33.3) | 0 | 0.15(0.07-0.84) | 1.906(0.16-3.66) |  |
|  | Normal | 321(82.7) | 25(89.3 | 3(50) | 0 | 0.15(0.78-1.7) | 0.44(0.79-1.6) |  |
|  | Above Average | 58(14.95) | 1(3.6) | 1(16.7) | 1(100 | 1 | 1 |  |
| History of chronic disease | Yes | 59(15.2) | 18(64.3 | 5(83.3) | 1(100 | **0.246(0.074-0.812)** | **2.56(1.78-3.4)** |  |
|  | No | 329(84.8) | 10(35.7 | 1(16.7) | 0 | 1 | 1 |  |

*Bold indicates statistically significant variable.

Table 10. frequency of individual menopausal symptoms among middle-aged women residing in Arba Minch DHSS, Ethiopia. 2022.

| Menopausal symptoms |  | Frequency | Percentage |
| --- | --- | --- | --- |
| Hot flush | Yes | 153 | 36.2 |
|  | No | 270 | 63.8 |
| Heart discomfort | Yes | 155 | 36.7 |
|  | No | 268 | 63.3 |
| Sleep problem | Yes | 175 | 41,4 |
|  | No | 248 | 58.6 |
| Depressive mood | Yes | 161 | 38 |
|  | No | 262 | 62 |
| Irritability | Yes | 166 | 39.2 |
|  | No | 257 | 60.8 |
| Anxiety | Yes | 29 | 6.8 |
|  | No | 394 | 93.2 |
| Physical and mental exhaustion | Yes | 33 | 7.8 |
|  | No | 390 | 92.2 |
| Sexual problem | Yes | 156 | 36.8 |
|  | No | 267 | 63.2 |
| Bladder problem | Yes | 82 | 19.4 |
|  | No | 341 | 80.6 |
| Vaginal dryness | Yes | 160 | 37.8 |
|  | No | 263 | 62.2 |
| Joint pain | Yes | 160 | 37.8 |
|  | No | 263 | 62.2 |
